# Facilitating access to pre-processed research evidence in public health

**DOI:** 10.1186/1471-2458-10-95

**Published:** 2010-02-24

**Authors:** Paula Robeson, Maureen Dobbins, Kara DeCorby, Daiva Tirilis

**Affiliations:** 1 Faculty of Health Sciences, McMaster University, 1200 Main St. W., Hamilton, Ontario, Canada

## Abstract

**Background:**

Evidence-informed decision making is accepted in Canada and worldwide as necessary for the provision of effective health services. This process involves: 1) clearly articulating a practice-based issue; 2) searching for and accessing relevant evidence; 3) appraising methodological rigor and choosing the most synthesized evidence of the highest quality and relevance to the practice issue and setting that is available; and 4) extracting, interpreting, and translating knowledge, in light of the local context and resources, into practice, program and policy decisions. While the public health sector in Canada is working toward evidence-informed decision making, considerable barriers, including efficient access to synthesized resources, exist.

**Methods:**

In this paper we map to a previously developed 6 level pyramid of pre-processed research evidence, relevant resources that include public health-related effectiveness evidence. The resources were identified through extensive searches of both the published and unpublished domains.

**Results:**

Many resources with public health-related evidence were identified. While there were very few resources dedicated solely to public health evidence, many clinically focused resources include public health-related evidence, making tools such as the pyramid, that identify these resources, particularly helpful for public health decisions makers. A practical example illustrates the application of this model and highlights its potential to reduce the time and effort that would be required by public health decision makers to address their practice-based issues.

**Conclusions:**

This paper describes an existing hierarchy of pre-processed evidence and its adaptation to the public health setting. A number of resources with public health-relevant content that are either freely accessible or requiring a subscription are identified. This will facilitate easier and faster access to pre-processed, public health-relevant evidence, with the intent of promoting evidence-informed decision making. Access to such resources addresses several barriers identified by public health decision makers to evidence-informed decision making, most importantly time, as well as lack of knowledge of resources that house public health-relevant evidence.

## Background

There is evidence demonstrating that the public health sector in Canada is working toward evidence-informed decision making (EIDM) [1-3], although there is still much work to be done to develop individual knowledge and skills, and organizational capacity to support, advance, and sustain it [4-6]. EIDM involves the translation of the best available evidence from a systematically collected, appraised, and analyzed body of knowledge [5]. It is defined as a process characterized by: 1) clearly articulating a practice-based issue; 2) searching for and accessing relevant evidence; 3) appraising methodological rigor and choosing the most synthesized evidence of the highest quality and relevance to the practice issue and setting that is available; and 4) extracting, interpreting, and translating knowledge, in light of the local context and resources, into practice, program and policy decisions [5,7]. Research evidence that is synthesized in a rigorous and transparent process provides more consistent and conservative estimates of effect [8-10] and therefore can be particularly powerful in informing and influencing public health policy and program decisions [11,12].

However, public health decision makers face considerable barriers in using relevant research evidence in policy and program decisions. Time is often cited as the number one barrier to EIDM experienced by public health professionals [13]. Human resource-related barriers such as the lack of sufficient staff with relevant knowledge and skill also exist [13,14]. Organizational barriers include: the lack of clearly communicated EIDM values; not including input from all levels in the organization in developing a strategic plan for EIDM; lack of leadership and champions; and inadequate resources and infrastructure to promote and support EIDM [13]. While initiatives are underway (e.g., health-evidence.ca, National Institute for Health and Clinical Excellence, Cochrane Public Health Group, and the Guide to Community Preventive Service of the Centers for Disease Control and Prevention) to increase the production and availability of synthesized, high quality research evidence relevant to public health practice, there continues to be significant challenges for public health decision makers in accessing this evidence when it exists, and many areas for which there is a lack of evidence evaluating the effectiveness of interventions (e.g., environmental health, social determinants of health, Aboriginal health [15-17].

The scope of public health practice includes prevention of chronic conditions, communicable diseases, and injury, as well as the protection and promotion of the health of populations. Thus, public health practitioners may be searching for evidence related to care, treatment, and prevention of a wide variety of conditions. As will be illustrated in this paper, public health decision makers currently must look to both clinical and public health-related resources to find relevant research evidence, given there is no one-stop-shop for public health-related evidence. While many resources appear at first glance to be clinically focused, for example Clinical Evidence, closer examination reveals that many public health-related documents are available through this resource. As such, all the resources described in this paper, even those better known for their clinical content, have been found to contain enough public health-relevant content to warrant it being identified as a source of evidence for public health decision makers.

**Figure 1:**
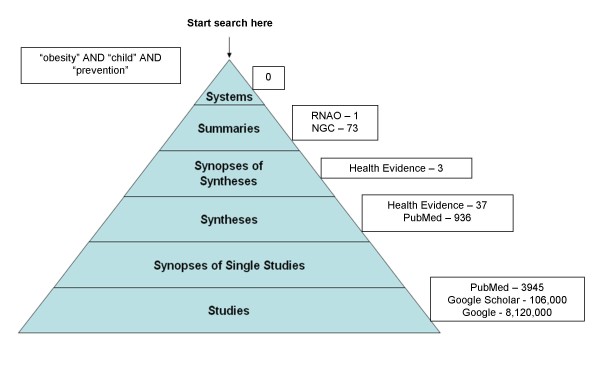
**Search results mapped to 6S pyramid**. Adapted from Accessing pre-appraised evidence: fine-tuning the 5S model into a 6S model, DiCenso, Bayley, & Haynes, 2009, 12, 99-101, 2010 with permission from BMJ Publishing Group Ltd.

The purpose of this paper is to: 1) describe a pyramid of pre-processed research evidence originally described by Haynes [18,19] and recently modified, with the addition of a sixth level by DiCenso, Bayley, and Haynes [20] (Figure 1); and 2) map to that pyramid those resources that house public health-relevant research evidence. This pyramid will reduce the time required by public health decision makers to seek out synthesized research evidence to inform policy and practice. As can be seen in Figure 1, the highest level of synthesized evidence is 'systems', followed by summaries, synopses of syntheses, syntheses, synopses of single studies, and finally single studies. The original and ongoing intent of classifying evidence in this way is to encourage decision makers to begin their search for evidence at the highest level of the pyramid, and therefore the most synthesized form of evidence, as opposed to beginning their search at the bottom of the pyramid, representing the least synthesized form of evidence. The pyramid can be used to guide decision makers in accessing the highest level of synthesized evidence and only moving to lower levels in the pyramid when no evidence exists at a higher level. It is our experience that public health decision makers generally start their search at the bottom of the pyramid and only come across more highly synthesized evidence by chance.

Two terms used throughout this paper require clarification before proceeding. The term "pre-processed" refers to evidence that has been synthesized and in some cases further summarized into key messages [21]. "Pre-appraised" refers to evidence that has been reviewed for methodological rigour and then in some instances is synthesized, and in other instances is both synthesized and summarized. While there are no internationally accepted criteria as to what constitutes a high quality resource that aims to facilitate access to pre-processed and/or pre-appraised evidence, at a minimum the following guiding principles can be used to assess the quality of the resources described in this paper: a) the process of filtering the evidence so that evidence of the highest quality is chosen for inclusion is transparent and rigorous; b) more than one person is involved in assessing the evidence for methodological quality and in synthesizing and summarizing it; c) the process for assessing, synthesizing, and summarising the evidence is explicit and rigorous; and d) the resource is updated regularly to ensure it remains current [20]. In this paper we provide a critique of only some of the better known resources identified at each level of the pyramid, using these four criteria. However, readers are encouraged to assess for themselves, the quality of the resources identified throughout this paper. The remainder of this paper describes each level of the pyramid and identifies resources at each level that house public health-relevant evidence.

## Methods

### 6S Pyramid of Pre-Processed Evidence

Table 1 identifies resources with public health content that exist for each level in the 6S pyramid, categorized according to whether they are publically and freely accessible or whether a subscription or other form of payment is required. All of the links were functioning at the time of publication. Table 2 summarizes our critique of the sites using the four criteria presented in the previous section.

**Table 1:** 6S Hierarchy of pre-processed evidence for public health

Levels of Haynes' pyramid	Publically available resources	Other resources
**Systems**	**none**	**none**

**Summaries**	**National Guidelines Clearinghouse (NGC) **http://guideline.gov **Guideline Advisory Committee (GAC) **http://www.gacguidelines.ca **National Institute for Health and Clinical Excellence Public Health Guidance **http://guidance.nice.org.uk/PHG?textonly=false **Registered Nurses Association of Ontario (RNAO) **http://www.rnao.org/bestpractices/index.asp **Trip Database **(filter by guidelines) http://www.tripdatabase.com **Canadian Medical Association **(CMA Infobase: clinical practice guidelines) http://www.cma.ca/index.cfm/ci_id/54316/la_id/1.htm **Alberta Medical Association **(Towards Optimized Practice) http://www.topalbertadoctors.org	**Clinical Evidence **http://www.clinicalevidence.com **Pier **http://pier.acponline.org/index.html **UpToDate **http://www.uptodate.com
**Synopses of Syntheses**	**Health Evidence **(**summary statements) **www.health-evidence.ca (pdf icon) **Effective Public Health Practice Project (EPHPP) **(all of these reviews and synopses are available through Health Evidence) **CDC Guide to Community Preventative Services **www.thecommunityguide.org **The Centre for Reviews and Dissemination (CRD) **http://www.crd.york.ac.uk/crdweb - **Database of Abstracts of Reviews of Effectiveness (DARE)** - **Economic Evaluation Database (NHS EED)** - **Health Technology Assessment (HTA) Database** **EPPI-Centre **http://eppi.ioe.ac.uk - **DoPHER**	**Evidence-based abstract journals** - Evidence-based Nursing http://ebn.bmj.com/search.dtl - Evidence-based Mental Health http://ebmh.bmj.com - Evidence-based Medicine http://ebm.bmj.com - Bandolier http://www.medicine.ox.ac.uk/bandolier/index.html - ACP (American College of Physicians) Journal Club www.acpjc.org **Evidence Digest** - a recurring column in *Worldviews on Evidence Based Nursing* **Evidence Updates **http://bmjupdates.com
**Syntheses**	**Health Evidence **www.health-evidence.ca **Effective Public Health Practice Project (EPHPP) **(Canada) (all of these reviews and synopses are available through Health Evidence) **CDC Guide to Community Preventative Services **www.thecommunityguide.org **The Cochrane Database of Systematic Reviews **http://www.cochrane.org/reviews/index.htm (Freely available in some jurisdictions; Relevant reviews included in the Health Evidence registry) **McMaster Health Knowledge Refinery** - **Public Health+ **http://www.nccmt.ca/tools/public_health_plus-eng.html - **McMaster PLUS Federated Search** - **Public Health PLUS (PH+) **http://www.nccmt.ca/tools/public_health_plus-eng.html - **Obesity+** - **Best Evidence for Nursing +** **AHRQ Evidence Reports **http://www.ahrq.gov/clinic/epcindex.htm **National Institute for Health and Clinical Evidence Public Health Guidance **http://guidance.nice.org.uk/PHG?textonly=false **The Campbell Collaboration (C2) **www.campbellcollaboration.org **The Centre for Reviews and Dissemination (CRD) **http://www.york.ac.uk/inst/crd/crddatabases.htm - **Database of Abstracts of Reviews of Effectiveness (DARE)** - **Economic Evaluation Database (NHS EED)** - **Health Technology Assessment (HTA) Database** **Evidence Based Health Promotion **http://www.health.vic.gov.au/healthpromotion/evidence_res/evidence_index.htm **PubMed **(using Clinical Queries feature with search filters for - ***systematic reviews ***http://www.ncbi.nlm.nih.gov/entrez/query/static/clinical.shtml#reviews - ***meta-analyses ***use the limits tab and click meta-analyses under publication type) **Trip Database **(filter by systematic reviews) http://www.tripdatabase.com	**The Cochrane Database of Systematic Reviews **http://www.cochrane.org/reviews/index.htm (requiring subscription in some jurisdictions) **Evidence Digest** - a recurring column in *Worldviews on Evidence Based Nursing* **McMaster Health Knowledge Refinery **http://hiru.mcmaster.ca/hiru/HIRU_McMaster_PLUS_projects.aspx - **ACP Journal Club Plus** - **Evidence Updates** - **BMJ Clinical Evidence**
**Synopses of Single Studies**	**PubMed Clinical Queries **www.ncbi.nlm.nih.gov/entrez/query/static/clinical.shtml **Trip Database **(filter by evidence-based synopses) http://www.tripdatabase.com **Evidence Updates **http://plus.mcmaster.ca/EvidenceUpdates/	**Evidence-based Nursing **http://ebn.bmj.com/search.dtl **Evidence-based Mental Health **http://ebmh.bmj.com **Evidence-based Medicine **http://ebm.bmj.com **Bandolier **http://www.medicine.ox.ac.uk/bandolier/index.html **ACP (American College of Physicians) Journal Club **www.acpjc.org
**Studies**	**McMaster Health Knowledge Refinery **http://hiru.mcmaster.ca/hiru/HIRU_McMaster_PLUS_projects.aspx - **Public Health PLUS (PH+) **http://www.nccmt.ca/tools/public_health_plus-eng.html - **Obesity+** - **Best Evidence for Nursing +** **PUBMED **(public interface for MEDLINE) www.pubmed.gov **Trip database **http://www.tripdatabase.com **Cochrane Central Register of Controlled Trials **(CENTRAL) http://www.mrw.interscience.wiley.com/cochrane/cochrane_clcentral_articles_fs.html (Freely available in some jurisdictions) **ClinicalTrials.gov **http://clinicaltrials.gov	**McMaster Health Knowledge Refinery **http://hiru.mcmaster.ca/hiru/HIRU_McMaster_PLUS_projects.aspx - **McMaster PLUS Federated Search** - **ACP Journal Club Plus** - **BMJ Clinical Evidence** **PUBMED **(public interface for MEDLINE) www.pubmed.gov **Trip database **http://www.tripdatabase.com **Cochrane Central Register of Controlled Trials **(CENTRAL) http://www.mrw.interscience.wiley.com/cochrane/cochrane_clcentral_articles_fs.html (Freely available in some jurisdictions) **ClinicalTrials.gov **http://clinicaltrials.gov

The resources and links in this document were active and accessible either freely or through payment as indicated at the time this paper was submitted for publication.

**Table 2:** Appraisal of evidence sources

Level and Type			Explicit Screening Process	Explicit & Rigorous Criteria for Assessing Quality, Summarizing, & Synthesizing	Multiple Independent Reviewers	Currency of the Site Content Easy to Determine
**Summaries**	Clinical Practice Guidelines	NGC	✓			✓ Site updated weekly, reviews current to within 5 years
		GAC	✓	✓	✓	✓ every 3 years minimum
		NICE	✓	✓	✓	unclear
		CMA Infobase	✓	✓		✓ Reviews current to within 5 years
		RNAO	✓	✓ Detail lacking regarding summarization and synthesis process		✓ every 3 years
		Towards Optimized Practice				
	Electronic Textbooks	Clinical Evidence	✓	✓	✓	Not clear; updating schedule varies by review
		UpToDate			✓	Reviews are revised as important new information is published.
		PIER			✓	✓
**Synopses of syntheses**		DARE	✓	✓ lacking in details on synthesis and summary	✓	✓ updated weekly
		Health Evidence	✓	✓	✓	unclear
		CDC	✓		✓	unclear
		Abstraction journals	✓	✓	✓	weekly
		EPPI-Centre	✓	✓	✓	Quarterly
**Syntheses**		AHRQ EPC	✓	✓	✓	unclear
		NICE	✓	✓	✓	unclear
		Cochrane	✓	✓	✓	Every two years
		C2	✓	✓	✓	Unclear
		CRD	✓	✓	✓	unclear
		PubMed Clinical Queries	✓	NA	NA	NA
		EPPI-Centre	✓	✓	✓	quarterly
**Synopses of single studies**		Abstraction journals	✓	✓	✓	
**Studies**		McMaster Knowledge refinery	✓	✓	✓	weekly
		PubMed Clinical Queries	✓	NA	NA	NA
		Bibliographic databases				

#### Systems

Systems are very detailed decision support services that match information from specific clients (individuals, groups, or populations) with the best available evidence that applies [18]. As described by Haynes [18,19,22] and DiCenso, Bayley, and Haynes [20], systems represent the ideal resource for EIDM as they contain all the research evidence about a specific client or population circumstance, linked to community health status reports and/or individual client records and to a synopsis of the existing relevant research literature with direct links to the original studies and/or reviews. While there are efforts currently underway to develop systems level data, it is not expected that a system such as this will be available for use in the near future. In presenting this level of evidence in their first publication of the pyramid, Haynes and colleagues, are primarily pointing out what the ideal level of evidence would be if it were feasible and obtainable. Furthermore, while systems level evidence may one day be available in the acute care sector, the challenges in creating a system such as this for public health likely make the possibility of realizing this level of evidence unlikely. Given the absence of such systems, public health decision makers must start at the next level of the pyramid.

#### Summaries

"Summaries integrate the best available evidence from the lower levels (drawing on syntheses [e.g., systematic reviews] as much as possible) to provide a full range of evidence concerning management options for a given health problem" [18]. Summaries, for example evidence-informed clinical practice guidelines and electronic text books, "can easily be made universally available (e.g., via the internet), and are more feasible to keep up to date and provide at least passive decision support by automatically linking them to individual patient and or community level problems" [18].

##### Clinical Practice Guidelines

Likely the most well known source for evidence-informed clinical practice guidelines is the National Guidelines Clearinghouse (NGC) http://guideline.gov. The NGC, an initiative of the Agency for Healthcare Research and Quality (AHRQ), is a repository of clinical practice guidelines that have been conducted by others. A search of this site using the term 'public health' identified 1337 guidelines. On brief examination many of these guidelines were directly relevant to public health practice (e.g. strategies to promote physical activity, emergency preparedness, management of Hepatitis B, and management of foodborne illnesses). While some quality measures are in place (see Table 2 for appraisal of site), the responsibility of assessing the methodological quality of the guidelines falls to the users.

Other sources of guidelines can be found through the Guideline Advisory Committee (GAC) http://www.gacguidelines.ca; the National Institute for Health and Clinical Excellence's Public Health Guidance http://guidance.nice.org.uk/PHG?textonly=false; the Canadian Medical Association (CMA Infobase: clinical practice guidelines) http://www.cma.ca/index.cfm/ci_id/54316/la_id/1.htm; the Registered Nurses Association of Ontario (RNAO) Best Practice Guidelines http://www.rnao.org/bestpractices/index.asp; and the Alberta Medical Association (Towards Optimized Practice) http://www.topalbertadoctors.org.

GAC is similar to the NGC in that it houses guidelines that have been conducted by others. Public health-related guidelines available on this site include those related to screening (cancer, infant hearing), obesity prevention, smoking cessation, contraception, and prenatal care. GAC is a high quality source of methodologically rigorous guidelines, and will be particularly useful for those involved in decision making in the topics listed above.

NICE provides national (UK) guidance regarding health promotion, injury and disease prevention, and treatment in four areas: public health, health technologies, interventional procedures, and clinical practice. This guidance is developed using the best available evidence and involving stakeholders in a transparent and collaborative manner. NICE produces two versions of its public health guidance: a guidance document that contains the recommendations and a summary of the related evidence; and a quick reference guide that provides an easy-to-read version of recommendations. While the process for making recommendations is rigorous and explicitly described on this site, it is unclear how often recommendations are updated. However, this is an important resource for public health effectiveness evidence.

The Canadian Medical Association Infobase: Clinical Practice Guidelines is similar to the NGC, in that it houses guidelines that have been conducted by others that meet specific inclusion criteria. CMA Infobase currently houses more than 1200 guidelines, several of which are related to chronic disease prevention, breastfeeding promotion, tobacco use, and prenatal care. Only those guidelines that are relatively current are made available on this site, although it is not clear how often guidelines are updated in order to remain on the site. While CMA Infobase is a good place to begin searching for guidelines, it is the user's responsibility to assess guidelines for quality.

The RNAO is involved in creating guidelines relevant to nursing practice and then making them easily accessible through their website. Of the 42 available guidelines approximately one-quarter are relevant for public health practice (e.g. breastfeeding, tobacco cessation, obesity prevention, intimate partner violence). The RNAO site is a good place to begin searching for evidence.

##### Electronic Textbooks

One electronic textbook that provides evidence relevant for health service decision making and an explicit description of the processes through which evidence is collected and appraised, is Clinical Evidence http://www.clinicalevidence.com. Owned by the BMJ Publishing Group Limited and requiring a subscription to access full-text, Clinical Evidence conducts systematic reviews using a thorough search strategy and rigorous process to assess the methodological quality of the literature. A search of the site using the term 'public health' identified 105 relevant reviews for a variety of public health topics including sexual health, physical activity, vaccines, obesity prevention, breastfeeding promotion, and smoking cessation. The term 'injury prevention' identified 110 reviews, and the term 'chronic disease prevention' identified 197 reviews. Clinical Evidence is a high quality site that houses and synthesizes many rigorous systematic reviews relevant to public health practice.

UpToDate http://www.uptodate.com is another evidence-based electronic textbook. Owned and operated by Wolters Kluwer Health, a leader in providing information and business intelligence to the healthcare field, UpToDate has more than 4000 physicians keeping over 7000 topics regularly updated. The intent of UpToDate is to review the best evidence to address questions that arise in clinical practice. While UpToDate is primarily clinically focused, areas of relevance to public health include vaccines, infectious diseases, screening, and prenatal and postnatal care. While the process for assessing methodological quality and synthesizing the evidence could be stronger, UpToDate is a useful resource for public health decision making.

PIER (Physicians' Information and Education Resource) http://pier.acponline.org provides timely evidence-informed guidance tailored to specific clinical topics. On the site's home page a list of updated modules with the date of the update is provided. While evidence is reviewed by experts using formal quality assessment criteria, the process could be more explicitly stated. While PIER has a clinical focus, topics relevant to public health include cancer and other chronic diseases, screening, falls prevention, pregnancy prevention, prenatal care, and smoking cessation.

#### Synopses of syntheses

Synopses of syntheses are "succinct descriptions of systematic reviews or meta-analyses" that aim to provide the right amount of evidence (not too much nor too little) to inform an intervention [18,19]. Ideally, these describe the research question, the study groups, the outcomes, and the measure of effect or other results of a body of evidence [22]. These summaries often discuss the methodological quality of the synthesis and the relevance of the findings to health practice, program development, and policies [20].

##### Databases

One example is the Database of Abstracts of Reviews of Effectiveness (DARE) of the Centre for Review Dissemination (CRD) http://www.crd.york.ac.uk/crdweb at the National Institute for Health Research in the United Kingdom http://www.nihr.ac.uk. DARE assists decision makers by systematically identifying and describing systematic reviews, appraising their quality and highlighting their relative strengths and weaknesses. A search of DARE identified 123 reviews on chronic disease prevention, 762 on health education, 126 on environmental health, 167 on sexual health, and 10 on communicable diseases. In addition to DARE, the CRD has additional databases such as the Economic Evaluation Database (NHS EED), and the Health Technology Assessment (HTA) Database, both of which include public health relevant reviews. This high quality site is an important resource for public health practice.

Health Evidence http://www.health-evidence.ca, a registry of published literature reviews evaluating the effectiveness of public health interventions, also provides short summaries of rigorous reviews [23]. A search of this site of 1900 reviews identified 586 reviews related to chronic disease prevention, 298 on addictions/substance use, 238 on communicable diseases, and 63 on environmental health. It is not clear however, how often the site is updated and summaries exist for only a small number of the reviews.

Another source of public health review evidence for which synopses are provided is the CDC Guide to Community Preventative Services http://www.thecommunityguide.org. The Community Guide has conducted systematic reviews on more than 200 interventions relevant to public health practice including adolescent health, alcohol, cancer, diabetes, HIV/AIDS, mental health, obesity, social environment, and vaccines. While a rigorous process is used in selecting, assessing methodological quality, and synthesizing evidence, it is unclear how often the site is updated.

Evidence for Policy and Practice Information and Co-ordinating Centre (EPPI-Centre) http://eppi.ioe.ac.uk is part of the Social Science Research Unit at the University of London. The EPPI-Centre conducts high quality systematic reviews relevant to public health practice and provides short summaries of these reviews. One of the databases located at this site, the Database of Promoting Health Effectiveness Reviews (DoPHER), contains 2500 public health relevant reviews and synopses. The site is updated quarterly.

##### Abstraction Journals

Evidence-based abstract journals such as *Evidence-Based Nursing*, *Evidence-Based Medicine*, *Evidence-based Mental Health*, *Evidence-Based Child Health*, and the *ACP Journal Club *provide synopses of syntheses. These synopses provide a summary of and clinical commentary about the related publication [19,22]. All of the journals listed here use a rigorous and explicit process for screening citations, assessing for methodological quality and in writing synopses. A search of Evidence-Based Nursing using the term obesity prevention identified 386 synopses, 192 related to screening, 1158 on maternal health, and 1006 on substance use prevention. Public health-relevant synopses were identified for all of the remaining abstraction journals.

#### Syntheses

Syntheses combine, using explicit and rigorous methods, the results of multiple single studies to provide a single set of findings, and include systematic reviews and meta-analyses [18,19,22,24]. As mentioned above Health Evidence houses syntheses specific to public health as well as CDC's Guide to Community Preventive Services. Additional sources of public health-related syntheses include the Evidence-based Practice Centers (EPC) Program of the Agency for Healthcare Research and Quality http://www.ahrq.gov/clinic/epcindex.htm, the Centre for Public Health Excellence at NICE; and the EPPI-Centre.

The EPCs use a rigorous process to conduct high quality systematic reviews. However, it is unclear how often reviews are updated. Reviews relevant to public health include screening for depression, breast cancer, and coronary heart disease, and other preventive health and emergency preparedness services. In addition to providing public health guidelines, NICE also conducts or commissions rigorous systematic reviews that inform their development. The EPPI-Centre also conducts high quality systematic reviews relevant to public health practice. Similar to other sites listed here while the process for conducting systematic reviews is rigorous and explicitly described, it is unclear how often reviews are updated.

Other sources of high quality syntheses, that house public health-relevant reviews include: The Cochrane Database of Systematic Reviews http://www.cochrane.org/reviews/index.htm, which is freely available in some jurisdictions; the Campbell Collaboration (C2) http://www.campbellcollaboration.org; and the Centre for Reviews and Dissemination (CRD) http://www.york.ac.uk/inst/crd/crddatabases.htm. Finally, PubMed, the public interface for MEDLINE, enables access to syntheses through built-in search filters for systematic reviews http://www.ncbi.nlm.nih.gov/entrez/query/static/clinical.shtml#reviews, by using the limits tab and clicking meta-analyses under publication type. While many public health reviews can be found in this way, it is the user's responsibility to assess the methodological quality of any identified reviews.

#### Synopses of Single Studies

Synopses of single studies are very brief descriptions of studies that aim to provide the right amount of evidence (not too much nor too little) to inform a public health intervention [18,19]. Ideally, these describe the research question, the study groups, the outcomes, and the measure of effect or other results of a body of evidence [22]. The same abstraction journals listed above for synopses of syntheses also provide synopses of single studies.

#### Studies

Sometimes, particularly in public health, the only available evidence is at the level of single studies [17]. Currently a number of federated search engines, such as the TRIP database http://www.tripdatabase.com, have been developed to enable users to search across multiple databases simultaneously. While a rigorous process has been used to include relevant databases it is the responsibility of the user to critically appraise whatever evidence is identified. Search results can be filtered to identify practice guidelines, synopses, systematic reviews and single studies. A quick search of TRIP using the term public health identified 1400 guidelines, 1203 synopses, 3300 systematic reviews, and over 26,000 single studies. Similar to the TRIP database, MacPlus Federated Search (FS) http://plus.mcmaster.ca/MacPLUSFS, searches multiple databases simultaneously and produces search results that are organized according to the 6S pyramid. However, MacPlusFS goes further by critically appraising the evidence, providing this assessment to users, and having health professionals rate the newsworthiness and relevance of the study to the field. MacPlusFS is one of a several such evidence services of the McMaster Health Knowledge Refinery (HKR) http://hiru.mcmaster.ca/hiru/HIRU_McMaster_HKR.aspx, a continuously updated resource for evidence-informed decisions [25]. It is currently available to any McMaster student, staff, faculty, or alumni upon registration for MacPLUS [25]http://hiru.mcmaster.ca/hiru/HIRU_McMaster_PLUS_projects.aspx. In addition to MacPluFS, the McMaster HKR includes ACP Journal Club Plus, PIER, Obesity+, Evidence Updates, and the related Public Health PLUS (PH+). In the case of PH+, which is accessible for free through the National Collaborating Centre for Methods and Tools, relevant articles from over 140 academic journals are critically appraised; those that are methodologically sound are identified and subsequently rated on a 7 point scale by public health decision makers for relevance and newsworthiness; and those receiving a rating of 4 or more are posted on the website, http://www.nccmt.ca/tools/public_health_plus-eng.html. Public health decision makers can sign up to receive alerts on new additions to the site or to become raters.

Other non-appraised evidence sources can be found through searches using traditional bibliographic databases such as MEDLINE, CINAHL, EMBASE, PsychInfo, Sociological Abstracts, Cochrane Central Register of Controlled trials (part of the Cochrane Library), Sport Discus, and ERIC. Such databases provide access to mainly published literature. PubMed http://www.pubmed.gov provides public access to Medline and user-friendly search tools including Clinical Queries http://www.ncbi.nlm.nih.gov/corehtml/query/static/clinical.shtml that includes built-in filters to enable quick location of relevant and high quality single studies [20].

### Application of the 6S Pyramid

A practical example will now be discussed to illustrate the pyramid's utility when used by a public health decision maker. A decision maker is searching for evidence to answer the question "Which strategies are effective in reducing obesity among children?" Using text based search terms "obesity AND child" and searching through known sources of public health evidence mapped to the 6S pyramid, the number of results identified at each level in the pyramid are depicted in Figure 1. At the bottom of the pyramid, the numbers (3945, 106,000, and 8,120,000 from PubMed, Google Scholar, and Google respectively) would be overwhelming to any searcher. However, as one rises up the pyramid, the number of documents found becomes more manageable (for example, at Health Evidence three synopses and 37 syntheses were located and 37 summaries in the form of practice guidelines were found between the RNAO and NGC sites). Of further benefit is that much of the work associated with the process of evidence-informed decision making (critical appraisal, synthesis, etc) has already been completed at the higher levels of the pyramid, saving decision makers time and other resources.

## Conclusion

This paper has described an existing hierarchy of pre-processed evidence and adapted it to the public health setting. A number of resources with public health-relevant content that are either freely accessible or requiring a subscription have been identified. This public health-relevant pyramid will facilitate easier and faster access to pre-processed, high quality public health evidence, with the intent of promoting evidence-informed decision making. Access to such resources addresses several barriers identified by public health decision makers to evidence-informed decision making, most importantly time, as well as lack of knowledge of resources that house public health-relevant evidence, and access to evidence that has been assessed for methodological quality, synthesized, and summarized. Enhanced access to high quality, synthesized research evidence is one component of a knowledge translation strategy to support and encourage evidence-informed public health practice, program, and policy decision making.

## Competing interests

The authors declare that they have no competing interests.

## Authors' contributions

PR identified, through her work with Health Evidence as a Knowledge Broker with public health decision makers in case studies, the need for the resource on which this paper is based; participated in the design of these studies; conducted the search for resources; mapped those resources to the levels of the 6S model; and drafted the manuscript. MD conceived of and designed the case studies being conducted to determine effective approaches to knowledge brokering for evidence-informed decision making in public health departments; helped to draft and edit the manuscript; and identified additional resources. KD coordinated the case studies; and participated in the design of these studies. DT assisted with the search for additional resources and the development of the final Health Evidence resource. All authors read and approved the final manuscript.

## Pre-publication history

The pre-publication history for this paper can be accessed here:

http://www.biomedcentral.com/1471-2458/10/95/prepub
